# Quantitative Trait Loci for Resistance to the Congenital Nephropathy in Tensin 2-Deficient Mice

**DOI:** 10.1371/journal.pone.0099602

**Published:** 2014-06-26

**Authors:** Hayato Sasaki, Nobuya Sasaki, Tomohiro Nishino, Ken-ichi Nagasaki, Hiroshi Kitamura, Daisuke Torigoe, Takashi Agui

**Affiliations:** 1 Laboratory of Laboratory Animal Science and Medicine, Department of Disease Control, Graduate School of Veterinary Medicine, Hokkaido University, Sapporo, Japan; 2 Laboratory of Laboratory Animal Science and Medicine, Faculty of Veterinary Medicine, Kitasato University, Towada, Japan; 3 Section of Biological Safety Research, Chitose Laboratory, Japan Food Research Laboratories, Chitose, Japan; 4 Department of Veterinary Physiology, School of Veterinary Medicine, Rakuno Gakuen University, Ebetsu, Japan; Institut National de la Santé et de la Recherche Médicale, France

## Abstract

The ICR-derived glomerulonephritis (ICGN) mouse is a chronic kidney disease (CKD) model that is characterized histologically by glomerulosclerosis, vascular sclerosis and tubulointerstitial fibrosis, and clinically by proteinuria and anemia, which are common symptoms and pathological changes associated with a variety of kidney diseases. Previously, we performed a quantitative trait locus (QTL) analysis to identify the causative genes for proteinuria in ICGN mice, and found a deletion mutation of the tensin 2 gene (*Tns2^nph^*, MGI no: 2447990). Interestingly, the congenic strain carrying the *Tns2^nph^* mutation on a C57BL/6J (B6) genetic background exhibited milder phenotypes than did ICGN mice, indicating the presence of several modifier genes controlling the disease phenotype. In this study, to identify the modifier/resistant loci for CKD progression in *Tns2*-deficient mice, we performed QTL analysis using backcross progenies from susceptible ICGN and resistant B6 mice. We identified a significant locus on chromosome (Chr) 2 (LOD = 5.36; 31 cM) and two suggestive loci on Chrs 10 (LOD = 2.27; 64 cM) and X (LOD = 2.65; 67 cM) with linkage to the severity of tubulointerstitial injury. One significant locus on Chr 13 (LOD = 3.49; approximately 14 cM) and one suggestive locus on Chr 2 (LOD = 2.41; 51 cM) were identified as QTLs for the severity of glomerulosclerosis. Suggestive locus in BUN was also detected in the same Chr 2 region (LOD = 2.34; 51 cM). A locus on Chr 2 (36 cM) was significantly linked with HGB (LOD = 4.47) and HCT (LOD = 3.58). Four novel epistatic loci controlling either HGB or tubulointerstitial injury were discovered. Further genetic analysis should lead to identification of CKD modifier gene(s), aiding early diagnosis and providing novel approaches to the discovery of drugs for the treatment and possible prevention of kidney disease.

## Introduction

ICR-derived glomerulonephritis (ICGN) mice are an inbred strain that exhibit glomerulosclerosis characterized by marked glomerular hypertrophy, mesangial expansion, foot process effacement of podocytes and thickening of the glomerular basement membrane. The ICGN mouse is also a chronic kidney disease (CKD) model that presents common symptoms and pathological changes associated with a variety of kidney diseases, such as albuminuria, hypoproteinemia, hyperlipidemia, anemia and systemic edema and eventual end-stage renal failure [1], [2]. In ICGN mice, nephrotic syndrome occurs at the suckling stage, which is unique in spontaneously nephropathic strains of mice or rats [3]. Although glomerular alteration is observed from two weeks after birth, renal function is preserved until three to four months after birth, when ICGN mice begin to show tubulointerstitial fibrosis [4]. This pathological process in ICGN mice conforms well with the clinical presentation of glomerular disease in humans [5].

Proteinuria is intimately involved in the dysfunction of podocytes or slit diaphragm, and glomerulosclerosis (GS) sequentially starts with podocytopenia. A line of evidence suggests that podocytes act as the main component of this barrier as mutations in a number of podocyte-specific genes (*NPHS1, NPHS2, CD2AP, ACTN4, TRPC6, PLCE1, MYH9*) responsible for GS have been identified [6], [7]. Therefore, podocyte loss is a common determining factor for progression toward many types of kidney disease [8], [9]. Previously, we performed a QTL analysis to isolate the causative genes for ICGN phenotypes, and identified a major QTL on chromosome (Chr) 15 that is identical to a single recessive locus causing albuminuria, and found a deletion mutation of the tensin 2 gene (*Tns2*, also known as *Tenc1*). This mutation (*Tns2^nph^*, MGI no: 2447990) leads to the loss of tensin 2 in podocytes, parietal epithelial cells and tubular epithelial cells [10]. The tensin (Tns) family of multidomain scaffold proteins (including Tns1, Tns2, Tns3 and Tns4) bind to the cytoplasmic tail of β-integrins, and localize to adhesions that anchor stress fibers in cells [11]. Therefore, Tns2 is thought to be a component of the actin cytoskeletal structures linking actin filaments with focal adhesions and plays a role as an intracellular signal transduction mediator through integrin [11]. It has been reported that Tns2 may be involved in the expression of nephrin (*Nphs1*) and synaptopodin (*Synpo*) in podocytes [12]. However, the mechanism by which *Tns2* deficiency leads to CKD is unclear.

In humans, the prevalence rates of CKD vary considerably by race, sex and age [13]. Multiple studies, such as genome-wide association studies, have identified many genetic factors that participate in kidney disease, although it is estimated that a vast amount of genetic factors for CKD remain to be identified [14]. CKD has a complex etiology resulting from the combination of multiple genetic and environmental factors, each with small effects. Epistatic interactions among susceptibility modifier loci make many of the loci difficult to detect in genome-wide association studies [15], [16]. Several reports using mouse models of nephropathy have demonstrated similar phenomena in that the effects of nephropathy-inducing factors were attenuated in other genetic backgrounds [17], [18], [19]. Recently, we have shown that congenic strains carrying the *Tns2^nph^* mutation on a B6 or 129×1/SvJ (129) genetic background exhibited milder phenotypes than do ICGN mice [20], [21]. In contrast, other researchers have reported DBA/2J (D2) congenic mice with *Tns2^nph^* were susceptible to CKD at a level similar to ICGN mice [22]. This suggests that B6 and 129 mice have resistant genes to CKD induced by *Tns2^nph^*. In the present study, we performed a genome-wide linkage analysis in (B6×ICGN) F_1_×ICGN backcross mice carrying *Tns2^nph^* to identify the CKD resistance genes and elucidate the mechanism by which *Tns2* deficiency leads to CKD.

## Materials and Methods

### Animals

ICGN mice were purchased from Osaka National Institute of Biomedical Innovation. C57BL/6J (B6: Charles River) mice were backcrossed to ICGN mice. *Tns2* (*nph*/*nph*) N_2_ backcross progenies were obtained by mating ICGN male mice to *Tns2* (+/*nph*) F_1_ hybrid female mice heterozygous for *Tns2^nph^*. Backcross mice homozygous for *Tns2^nph^* were born in accordance with Mendelian inheritance and at an equal sex ratio. B6-*Tns2^nph^* congenic mice were generated as described previously [20]. The animal room was air-conditioned at 22±4°C, maintained at 40–60% relative humidity, and mice were maintained under a 12 hr light-dark cycle. A standard laboratory diet, CE-2 (Nihon Clea, Tokyo, Japan), and tap water were available *ad libitum*. In this study, 16-week-old mice were selected for all analyses as the ICGN mice show severe albuminuria and anemia, which are the end stages of a variety of pathological conditions. A humane end point was applied when the mice with severe anemia became moribund.

### Ethical Statement

All research and experimental protocols were adhered to the Regulation for the Care and Use of Laboratory Animals, Hokkaido University and approved by the President of Hokkaido University following to the review of the Institutional Animal Care and Use Committee (Approval ID: No. 110226).

### Phenotypic Analysis

Blood samples were collected from the postcava under isoflurane anesthesia. Hemoglobin concentration (HGB), hematocrit (HCT) and blood urea nitrogen (BUN) were measured as described previously [20].

Formalin-fixed and paraffin-embedded kidney blocks were sectioned at a thickness of 2 µm, and stained with Periodic acid-Schiff (PAS). In this study, two types of histopathological scores were adopted to quantitate the severities of renal damage. A glomerular index, in which the severities were based on glomerular injury, was calculated from the scores for twenty glomeruli randomly selected from each sample. The severity of glomerular injury was scored as follows: 0, no abnormality or nearly normal; 1, expansion of the mesangial matrix; 2, severe expansion of the mesangial matrix; 3, hyalinosis or collapse (Fig. S1). First, the highest and lowest scores were removed to exclude bias in random selection. The sum of the eighteen glomerular scores was then taken as the glomerular index. However, if there were glomeruli that scored 3 points, the index was the sum of 36 and the number of glomeruli that scored 3 points. This calculation method means that a kidney with hyalinosis or collapse has a more severe glomerular injury than a kidney with only mesangial expansion. The tubular index was the sum of two histopathological parameters, urinary cast and tubule dilation. The severity of tubule dilation was performed using scale 0 to 2∶0, no abnormality; 1, mild; 2, severe. One point was then added to the tubular index if a urinary cast was detected (Fig. S2). These analyses were performed by two independent observers. There were remarkable congruences in both blinded scores [the interclass correlation coefficients (ICCs) = 0.96].

### Genotyping Analysis

We used 246 (male = 124, female = 122) *Tns2* (*nph*/*nph*) N2 backcross mice for a genome-wide scan. Extraction of genomic DNA from tail tips was performed by a standard method. *Tns2* genotypes were determined by PCR using genomic DNA from the tail tips. *Tns2^nph^* homozygous mice were identified using the primers AGACACCACCAGCACCTTCT and CGATCCAGCTCCTGTCTTTC, which distinguish the *Tns2^nph^* allele by the 8-base deletion in exon 18 of *Tns2* [10]. For QTL analysis, a total of 92 informative microsatellite markers and coat color were used for the genotyping analysis, as listed in Table S1. The map positions of the microsatellite markers were based on information from the Mouse Genome Informatics of Jackson Laboratory (MGI; http://www.informatics.jax.org/, MGI_4.41). PCR was carried out with a cycling sequence of 95°C for 5 min (one cycle), followed by 35 cycles consisting of denaturation at 95°C for 30 sec, primer annealing at 55°C for 30 sec, and extension at 72°C for 30 sec. Amplified samples were electrophoresed with 12% polyacrylamide gels and stained with ethidium bromide. The stained gels were then visualized and photographed under an ultraviolet lamp.

### QTL Analysis and Statistics

QTL analysis was performed using the Map Manager QTXb20 software program that uses a maximum likelihood algorithm with “interval mapping” and “simultaneous search”, and permits better localization of loci and exclusion mapping [23]. Recombination frequencies (%) were converted into genetic distance (cM) using the Kosambi map function. This program provides linkage data as a likelihood ratio statistic (LRS) score. For each Chr, the LRS values were calculated by 10000 random permutations of the trait values relative to the genotypes of the marker loci. Genome-wide significance thresholds were calculated in terms of LRS by 10000 random permutation tests based on the established guidelines. The logarithm of odds (LOD) score was calculated by dividing the LRS value by 4.61. LOD values of the suggestive and significant thresholds for each trait are summarized in Table 1. Significant linkage was determined according to the traditional threshold (LOD score >3.3) [24]. Statistical analyses were performed using the Stat-View program (SAS Institute Inc. Cary, USA). Spearman’s rank correlation analysis was used to find the correlation between two sets of tested traits.

**Table 1:** QTL identified for nephropathic traits.

Chr	Peak (cM)	Nearest marker	LOD	Phenotype	ICGN/ICGN^a^	B6/ICGN^a^	Resistance allele
2	31	*D2Mit369*	5.36	Tubule	1.0	0.4	B6^b^ **
2	36	*D2Mit380*	4.47	HGB (g/dL)	12.6	13.2	B6^c^
			3.58	HCT (%)	43.1	45.2	B6^c^
2	51	*D2Mit66*	2.41	Glomeruli	30.2	25.9	B6^b^ **
			2.34	BUN (mg/dL)	38.2	31.3	B6^c^
10	64	*D10Mit271*	2.27	Tubule	0.8	0.4	B6^b^ *
13	14	*D13Mit60*	3.49	Glomeruli	30.4	24.9	B6^b^ **
X	67	*DXMit186*	2.65	Tubule	0.8	0.4	B6^b^ *

a Data resent the mean of the phenotype.

b Mann-Whitney’s U test,

**P<0.001;

*P<0.01.

c Student’s T test, P<0.001.

## Results

### *1.* Phenotypic Characterization of *Tns2^nph^* Backcross Mice

**Figure 1:**
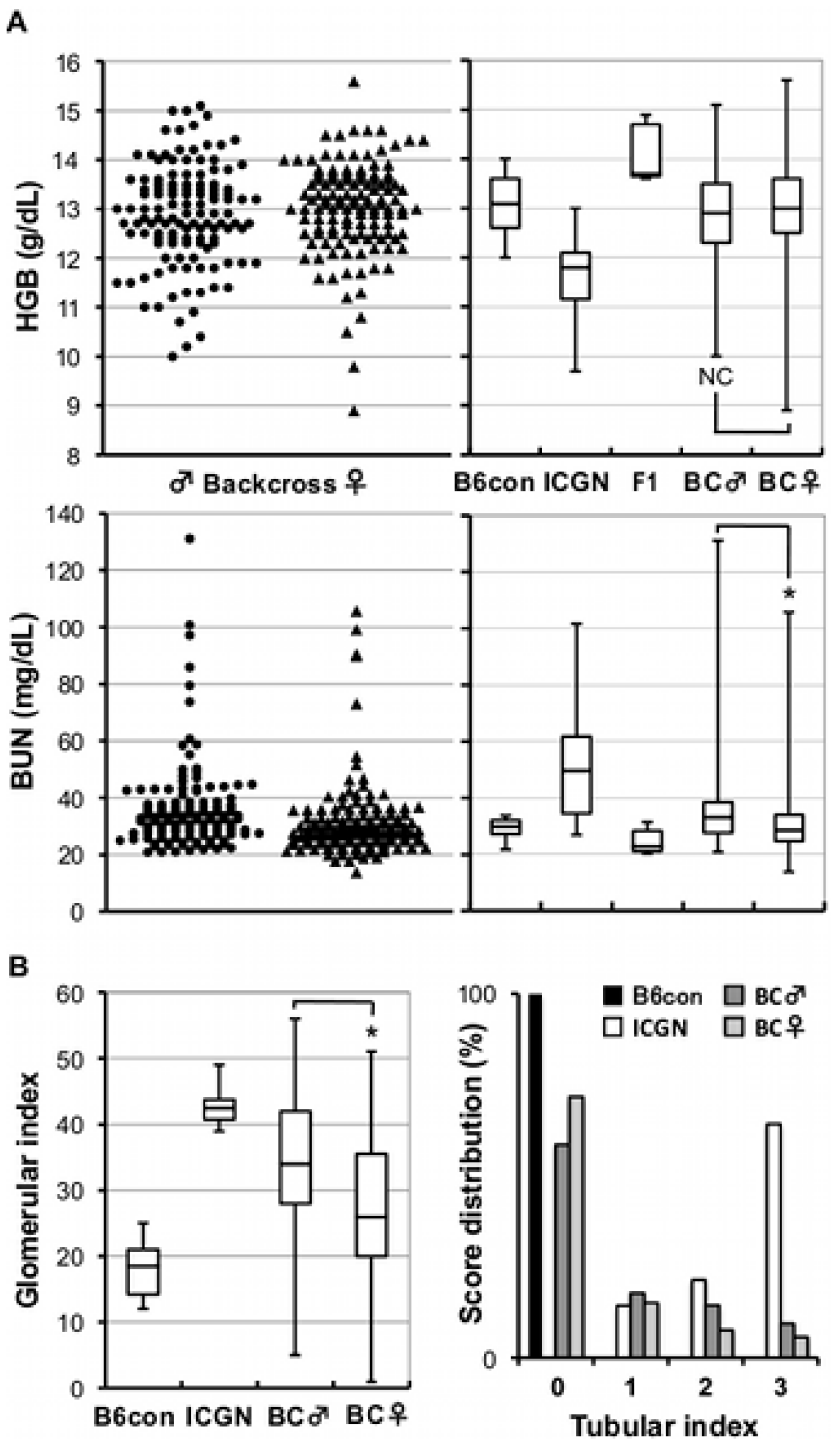
Hemoglobin concentration and BUN and kidney injury in backcross mice. (A, left) Distributions of HGB and BUN in (ICGN×B6) F_1_×ICGN backcross progenies. (A, right) Distributions of HGB and BUN in B6-*Tns2^nph^*, ICGN, (ICGN×B6) F_1_, backcross mice. (B) Glomerular index and tubular index distributions in (ICGN×B6) F_1_×ICGN backcross progenies. B6con, B6-*Tns2^nph^*; BC, backcross mice; NS, no significant; asterisk, *P*<0.001.

To verify the effect of genetic background on susceptibility to CKD induced by *Tns2^nph^*, we produced a total of 246 (ICGN×B6) F_1_×ICGN backcross mice and performed blood testing and histopathological analysis at the beginning of a genome-wide linkage analysis. Blood samples collected at 16 weeks old were analyzed. In CKD, a high BUN level and low HGB signify renal impairment and renal anemia, respectively, which is also a common symptom in ICGN mice (Fig. 1A, right). In backcross progenies, wide spectrums of BUN and HGB were observed from the various degrees of B6 type to that of ICGN types (Fig. 1A). These results imply the existence of multiple modifier loci for CKD caused by *Tns2^nph^*. In addition, the distribution of BUN in backcross males, as compared with females, was statistically more severe (P<0.001, Mann-Whitney’s U test).

Figure 1B shows the box plot for glomerular index and the histogram for tubular index. The scores histologically evaluate glomerular injury and tubulointerstitial injury, respectively. The backcross mice showed wide range of the histopathological severity, from B6 types to ICGN types (see also Fig. S1, S2, S3). There were also significant sexual dimorphism in both scores, with males exhibiting a higher level of severity than females (glomerular index P<0.001, tubular index P<0.05, Mann-Whitney’s U test). These differences were presumably due to the protective effect of estrogen in the female glomeruli [25].

The symptom severity of all tested traits exhibited significant correlations with each other according to Spearman’s rank correlation analysis (*P*<0.01). However, the Spearman’s rank correlation coefficients were comparatively smaller for BUN combinations (|r| = 0.27 ∼ 0.48) than for the other sets (|r| = 0.4 ∼ 0.65).

### *2.* Genome-wide Linkage Analysis for the Mapping of CKD-Resistant Genes in Backcross Mice

**Figure 2:**
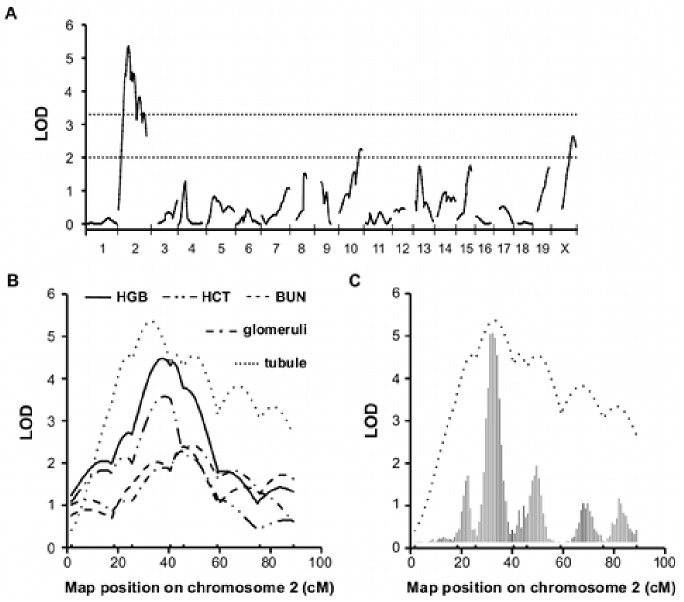
Linkage of nephropathy to chromosome 2. (A) Genome-wide linkage analysis of the tubular index. (B) LOD plots show the linkage of nephropathic traits to chromosome 2. HGB (solid line) and tubular index (dotted line) yielded significant LOD scores. The approximate 95% confidential intervals: 19.6–56.4 cM (tubule), 26–73 cM (glomeruli), 26–55 cM (HGB), 23–59 cM (HCT), 27–72 cM (BUN). (C) LOD curve and bootstrap histogram of the QTL on chromosome 2 for tubular index.

To identify the modifier genes for CKD, we carried out a genome-wide linkage analysis using *Tns2^nph^* backcross mice. HGB, HCT, BUN, glomerular index and tubular index at 16 weeks of age were used as indicators for CKD. Table 1 summarizes the microsatellite markers linked to the phenotype, LOD score, interval and phenotypic values of each genotype. The QTL analysis of the tubular index, which shows the intensity of tubulointerstitial injury, identified one significant locus on Chr 2 and two suggestive loci on Chrs 10 and X, respectively (Fig. 2A, Table 1). The significant QTL had a peak LOD score of 5.36 and explained 10% of the variance. Regarding the other nephropathic traits (HGB, HCT, BUN and glomerular index), significant or suggestive loci were also detected on Chr 2 (Fig. 2B, Table 1). Within these QTLs, each trait exhibited CKD resistance associated with the ICGN/B6 genotype.

**Figure 3:**
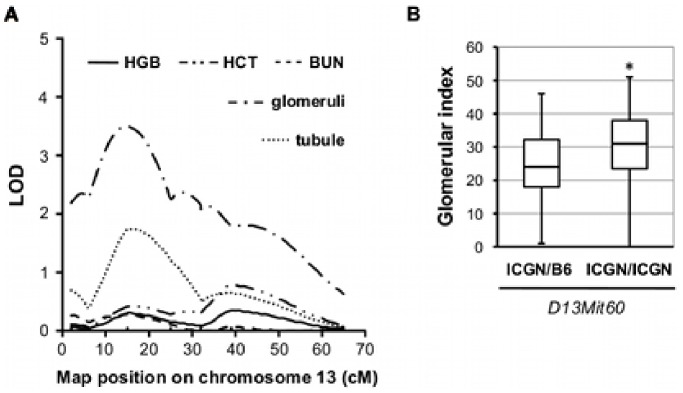
LOD score curves on chromosome 13. (A) A significant QTL for glomerular index was detected on chromosome 13. The approximate 95% confidential intervals: 0–31.5 cM. (B) Glomerular index at the peak LOD score (*D13Mit60*). The resistance phenotype associated with ICGN/B6 genotype (*P*<0.001, Mann-Whitney’s U test).

The QTL analysis of the glomerular index, which shows the severity of GS, identified one significant locus (peak LOD = 3.49) on Chr 13 (Fig. 3A). Grouping backcross mice by their genotype at the marker near the linkage peak, *D13Mit60* (14.44 cM), ICGN/B6 heterozygous mice showed statistically milder glomerular injury than did ICGN/ICGN homozygous mice (*P*<0.001, Mann-Whitney’s U test, Fig. 3B). Although the linkage of the tubular index to Chr 13 was similar to that of glomerular index, the peak LOD score was only 1.73. No suggestive linkages were observed for hematological phenotypes on Chr 13 (Fig. 3A).

**Figure 4:**
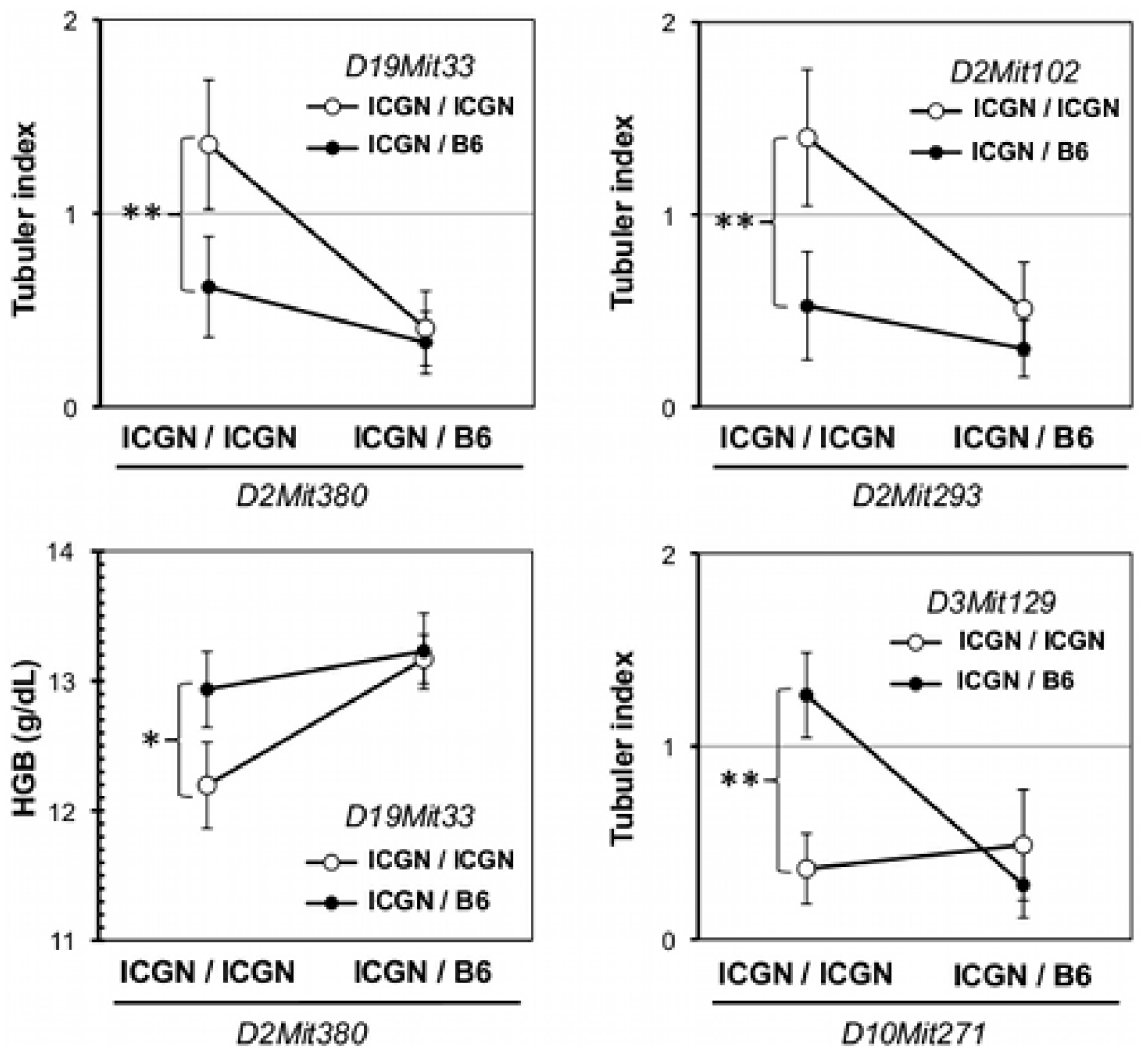
Epistasis associated with QTLs. Epistatic interactions were detected associated with the markers on chromosome 2 and 10. Open circles and closed circles represent homozygous and heterozygous variants for the tested markers, respectively. Asterisks signify significant differences between the two genotypes (**P<0.001; *P<0.01; Mann-Whitney’s U test).

Epistatic interactions were searched between all of the tested markers by MapManager QTXb20 (*P*<0.000001). We detected three significant interactions in the tubular index associated with markers on Chrs 2 and 10, which showed significant or suggestive linkages to tubular index (*P*<0.01, ANOVA test, Fig. 4). Genotype variations in the tubular index at *D2Mit380* (40.89 cM), *D2Mit293* (17.24 cM) and *D10Mit271* (72.31 cM) were only expressed in the presence of the ICGN/ICGN genotype at *D19Mit33* (51.76 cM), the ICGN/ICGN genotype at *D2Mit102* (57.65 cM) and the ICGN/B6 genotype at *D3Mit129* (80.49 cM), respectively. The B6 allele at *D19Mit33* also suppressed the genotypic effect of *D2Mit380* in HGB. *D2Mit293* (17.24 cM), which lies outside the 95% confidence interval for the QTL of the tubular index (19.6–56.4 cM), showed a significant epistatic interaction with *D2Mit102*, whereas the inner marker *D2Mit369* (24.51 cM) did not, although both markers exhibited significant LOD scores of 3.6 and 4.4, respectively. On the other hand, *D2Mit380* (40.89 cM) showed a significant epistatic interaction with *D19Mit33*, as did *D2Mit66* (49.45 cM) and *D2Mit102* (57.65 cM) (data not shown). These results suggest multiple QTLs for tubular index on Chr 2. The bootstrap analysis also supports this hypothesis (Fig. 2C). Next, to identify the candidate genes, we screened the QTLs on Chrs 2 and 13 for podocyte-related genes using XPodNet, and protein-protein interactions in the podocytes [19]. In total, 46 candidate genes are listed (Table 2 and 3).

**Table 2:** Podocyte-related genes on Chr 2 QTL.

Candidate genes	Interacting proteins (XPodNet)
*Enkur*	Pak1, Pik3r2
*Apbb1ip*	Enah, Vasp
*Pax8*	Paxip1, Wt1 (Chr 2∶55 cM)
*Grin1*	Ctnnb1, Src (Chr 2∶78 cM), Actb, Spnb2, Prkce, Camk2a, Camk2b, Dnm1 (Chr 2∶22 cM), Ppp3ca
*Notch1*	Wwp1, Psen1, Psen2, Ep300, Rbpj, Wdr12, Fbxw7, Kat2a, Kat2b, Smarcd3
*Vav2*	Lcp2, Rac1, Nckipsd, Ptpn6, Src (Chr 2∶78 cM)
*Rxra*	Trim24, Rarb, Rara, Ppara, Thra, Thrb, Zfp423, Nsd1, Med25
*Olfm1*	N.D.
*Tsc1*	Ezr, Ywhab (Chr 2∶85 cM)
*Abl1*	Nck1, Pxn
*Lamc3*	Nid1, Nid2, Lama5 (Chr 2∶103 cM)
*Dnm1*	Atp9a (Chr 2∶89 cM), Grin1 (Chr 2∶17 cM), Grin2b, Sh3gl2, Itsn1, Pfn2, Prnp (Chr 2∶64 cM), Ctsl, Ap2a2, Kif2a, Kif5a, Tuba1a
*Eng*	Tgfbr1, Tgfbr2
*Cdk9*	Cdkn1a, Cdkn1b
*Angptl2*	N.D.
*Lmx1b*	Ldb1, Tcf3, Bmpr2
*Mapkap1*	Akt1
*Crb2*	N.D.
*Lrp1b*	Pick1
*Zeb2*	Smad2
*Prpf40a*	Wasl
*Pkp4*	Ctnnb1
*Pla2r1*	N.D.
*Itga6*	Lama5 (Chr.2∶103 cM), Itgb1, Itgb4, Nisch
*Wipf1*	Cttn
*Hoxd1*	Ldb1 (Chr.19∶38.75 cM)
*Itgav*	Itgb1, Itgb3, Itgb5, Spp1, Plaur, Col4a3
*Ctnnd1*	Yes, Cdh2, Cdh4 (Chr.2∶102 cM), Cdh22 (Chr.2∶85 cM)
*Lrp4*	Pick1
*Syt13*	Nrxn1
*Wt1*	Pawr, Wtip, Fhl2, Fhl3, Pax2 (Chr.19∶38 cM), Pax8 (Chr.2∶16 cM)
*Pax6*	Paxip1

N.D.: not detected.

**Table 3:** Podocyte-related genes on Chr 13 QTL.

Candidate genes	Interacting proteins (XPodNet)
*Net1*	Magi1
*Actn2*	Camk2a
*Nid1*	Lama2, Lamc1, Lamc2, Lamc3, Hspg2, Eln
*E2f3*	Ccnd1
*Agtr1a*	Agtrap
*Ripk1*	Atg5
*Nm1*	N.D.
*Bmp6*	Bmpr1a
*Nedd9*	Src, Nck2, Ptk2
*Gadd45g*	Cdkn1a
*Nsd1*	Rxra
*Slc34a1*	Itgav
*Dbn1*	Gja1, Lrrk2
*Ntrk2*	Cdk5, Cdk5r1

N.D.: not detected.

We also detected seven genetic loci associated with blood cell traits (Table S2). These loci (except for Chr 2) were not linked to the histopathological traits, so that the loci only influenced hematological parameters between the two mice, independent of CKD. Correspondingly, numerous genetic loci of this kind have been identified in recent genome-wide linkage analyses of humans and other mouse strains [26], [27], [28], [29].

## Discussion

In this study, we report the mapping of genetic loci responsible for the resistance to congenital nephropathy caused by *Tns2* deficiency in a B6 genetic background. Analysis of backcrossing between resistant B6 mice and susceptible ICGN mice identified significant QTLs on Chrs 2 and 13. Among the tested traits, tubulointerstitial injury (tubular index) and anemia (HGB and HCT) were strongly associated with the same locus on Chr 2. In patients with CKD, anemia develops due to decreased renal erythropoietin synthesis resulting from tubulointerstitial damage. A significant locus for tubular index, ranging approximately from 19.6 to 56.4 cM on Chr 2, which overlaps the QTLs of the other tested traits, appears to be separated into three QTLs with peaks at 22, 31 and 50 cM (Fig. 2). The proximal QTL (22 cM) has epistatic interactions with *D2Mit102* and *D2Mit282* (Fig. 4, data not shown). The region between the two markers exhibiting a peak LOD score of 3.8 in tubular index includes the syntenic locus for albuminuria previously reported in humans, rats and mice [30], [31]. The middle QTL (31 cM) maps to the same region that was found in a (B6×D2) F_2_ intercross and in a (B6×A/J) F_2_ intercross as a second QTL for albuminuria on Chr 2 [30], [31]. The distal QTL (50 cM), where the glomerular score and BUN showed a suggestive linkage, maps to the same region as the suggestive locus for proteinuria that was found in an HIV-associated nephropathy (HIVAN) model mouse on a mixed genetic background of B6 and FVB [32]. The syntenic human region (2q32.1) is associated with the early onset of end-stage renal disease in black families enriched for nondiabetic nephropathy [33]. Taken together, the resistance to CKD in Tns2-deficient B6 mice can be mainly exerted in combination with the multiple loci on Chr 2.

While *Tns2* mRNA is broadly expressed in the kidney and other organs, significant abnormalities (i.e., podocytopenia) were only observed in the kidney of ICGN mice [1]. In the glomeruli, Tns2 expression is localized in podocytes [10], [34]. Therefore, the dysfunction of Tns2 in podocytes has been speculated to cause renal dysfunction. Tns is an actin-binding protein that is present in focal adhesions and has been suggested to play an important role in the actin cytoskeleton and integrin-mediated signaling [11]. Recently, it has been reported that Tns2 may bind to integrin β1, and regulate the activity of the integrin β1 downstream effector molecule, integrin-linked kinase (ILK), which is known to play an essential role in GBM assembly and podocyte function [22]. In addition, ILK expression is stimulated in podocytes by renal injury and induces podocyte epithelial-mesenchymal transition [35]. Interestingly, the podocyte-specific knockout of ILK induces progressive focal segmental GS with increased mesangial matrix deposition and downregulation of *Nphs1* and *Synpo* with age, as observed in ICGN mice [12], [36], [37]. Activation of FAK, another effector molecule of the integrin β1 signaling pathway, is also observed in the glomeruli of *Tns2* mutant mice and conditional *Ilk* knockout mice [22], [37]. This upregulation of FAK activity is considered to compensate for the ILK abnormality structurally, but not functionally [22], [37]. It is notable that the *Ilk* knockout genetic background is derived from B6 (*NPHS2* promoter-Cre transgenic mice) and 129 (ILK*^flox^* ^/*flox*^ mice) [36], [37]. That is, the genetic backgrounds of B6 and 129 are resistant to *Tns2*-deficiency, but their resistance has no effect on *Ilk*-deficiency. Hence, it is assumed that the resistance effects are exhibited by functionally regulating the disorders in the integrin β1-ILK signaling pathway or structurally reinforcing the fragility of the actin cytoskeleton and slit diaphragm induced by aberrant ILK activity in *Tns2*-deficient podocytes.

Under this assumption, we screened QTLs mapped on Chr 2 for podocyte-related genes using XPodNet and identified 32 candidate genes (Table 2). Among these, we were especially interested in *Notch1*, *Dnm1*, *Lmx1b*, *Zeb2* and *Wt1*. NOTCH1 (18.91 cM) is a transmembrane receptor protein whose signaling pathway correlates with GS and renal function [38]. DNM1 (22.09 cM), dynamin1, participates in endocytosis in the podocyte foot process and contributes to maintenance of the slit diaphragm [39]. WT1 (55.06 cM) and LMX1B (22.48 cM) are transcription factors that regulate podocyte-specific structural genes, *Nphs1* and *Podxl*, and *Nphs2* respectively, and play a key role in the development and maintenance of the actin cytoskeleton and slit diaphragm in podocytes [40], [41], [42]. ZEB2 (27.31 cM), a transcriptional repressor of E-cadherin, is upregulated by the integrin β1-ILK signaling pathway downstream target SNAIL and induces the epithelial–mesenchymal transition [43].

In the same way, 14 candidate genes of the Chr 13 QTL are listed in Table 3. This locus partially overlaps the HIVAN susceptibility locus, *HIVAN2*, that was found in an (HIV-1 transgenic FVB×B6) F_2_ intercross [32]. An expression quantitative trait locus (eQTL) analysis has revealed that *HIVAN2* influences the transcript abundance of *Nphs2*, a membrane protein linking the slit diaphragm to the cytoskeleton [32]. However, *HIVAN2* includes none of transcription factors known to regulate *Nphs2* expression. In our cross, the locus was not linked to anemia, so that it is not considered to be essential for susceptibility to CKD induced by *Tns2^nph^*.

Our genomic analysis of CKD in a cross between B6 and ICGN assumes that the Chr 2 loci critically contribute to resistance in *Tns2*-deficient mice. The Chr 2 QTLs identified in crosses for proteinuria between resistant B6 and susceptible D2 or A/J or HIV-1 transgenic FVB are similar to ours [30], [31], [32]. This suggests that our QTL is not limited to resistance to the CKD induced by *Tns2^nph^*, but is generally associated with resistance to CKD. Although this locus has not been noted in previous reports due to the absence of a homologous human QTL, we believe that this locus also plays an important role in resistance to CKD. Identification of resistant genes on this locus would help reveal the mechanism by which *Tns2* deficiency leads to CKD and provide a novel insight into susceptibility to CKD, leading to the development of treatment strategies for CKD.

## Supporting Information

Figure S1 **Representative examples of glomerular score.**
(TIF)

Figure S2 **Representative examples of tubular score.**
(TIF)

Figure S3 **Histopathological severity, resistant strain vs. susceptible strain.**
(TIF)

Table S1 **Genotyping markers.**
(XLSX)

Table S2 **QTLs identified for hematological phenotypes.**
(PPTX)
