# Supplementary figures and images for: Quantitative Trait Loci for Resistance to the Congenital Nephropathy in Tensin 2-Deficient Mice
Source: PLoS One. 2014 Jun 26;9(6):e99602. doi: 10.1371/journal.pone.0099602 (PMC4072594; doi:10.1371/journal.pone.0099602)

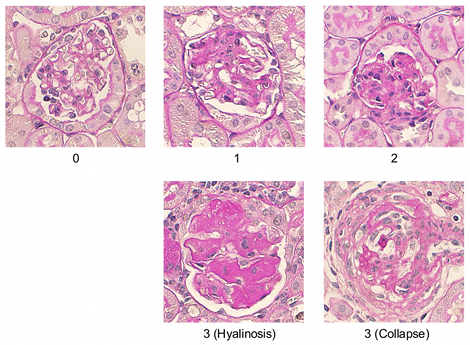

Supplement: Figure S1 — Representative examples of glomerular score. (TIF) [file pone.0099602.s001.tif]

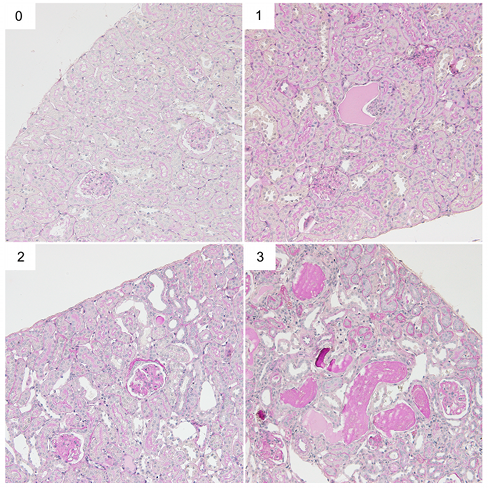

Supplement: Figure S2 — Representative examples of tubular score. (TIF) [file pone.0099602.s002.tif]

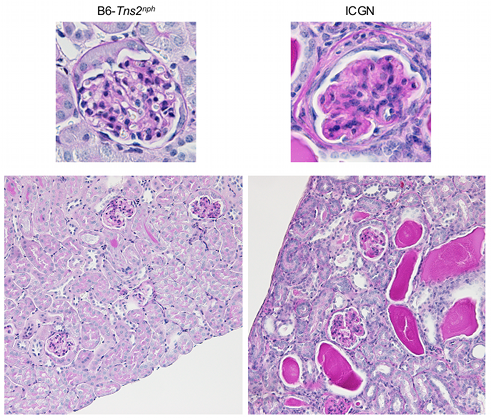

Supplement: Figure S3 — Histopathological severity, resistant strain vs. susceptible strain. (TIF) [file pone.0099602.s003.tif]
