# Supplementary material for: Quantitative Trait Loci for Resistance to the Congenital Nephropathy in Tensin 2-Deficient Mice
Source: PLoS One. 2014 Jun 26;9(6):e99602. doi: 10.1371/journal.pone.0099602 (PMC4072594; doi:10.1371/journal.pone.0099602)
Supplement: Table S2 — QTLs identified for hematological phenotypes. (PPTX) [file pone.0099602.s005.pptx]

## Slide 1
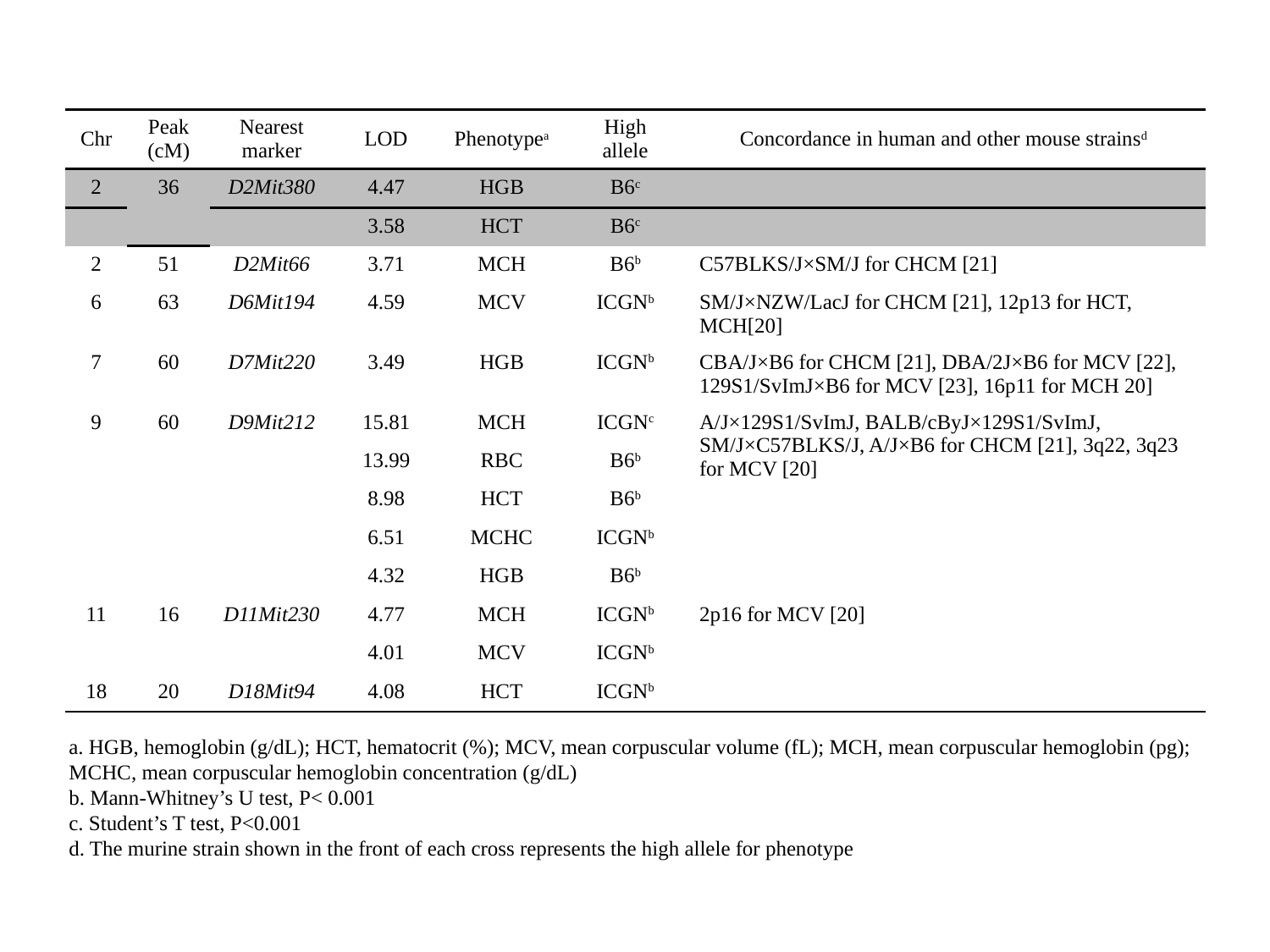

| Chr | Peak (cM) | Nearest marker | LOD | Phenotypea | High allele | Concordance in human and other mouse strainsd |
| --- | --- | --- | --- | --- | --- | --- |
| 2 | 36 | D2Mit380 | 4.47 | HGB | B6c | |
| | | | 3.58 | HCT | B6c | |
| 2 | 51 | D2Mit66 | 3.71 | MCH | B6b | C57BLKS/J×SM/J for CHCM [21] |
| 6 | 63 | D6Mit194 | 4.59 | MCV | ICGNb | SM/J×NZW/LacJ for CHCM [21], 12p13 for HCT, MCH[20] |
| 7 | 60 | D7Mit220 | 3.49 | HGB | ICGNb | CBA/J×B6 for CHCM [21], DBA/2J×B6 for MCV [22], 129S1/SvImJ×B6 for MCV [23], 16p11 for MCH 20] |
| 9 | 60 | D9Mit212 | 15.81 | MCH | ICGNc | A/J×129S1/SvImJ, BALB/cByJ×129S1/SvImJ, SM/J×C57BLKS/J, A/J×B6 for CHCM [21], 3q22, 3q23 for MCV [20] |
| | | | 13.99 | RBC | B6b | |
| | | | 8.98 | HCT | B6b | |
| | | | 6.51 | MCHC | ICGNb | |
| | | | 4.32 | HGB | B6b | |
| 11 | 16 | D11Mit230 | 4.77 | MCH | ICGNb | 2p16 for MCV [20] |
| | | | 4.01 | MCV | ICGNb | |
| 18 | 20 | D18Mit94 | 4.08 | HCT | ICGNb | |
a. HGB, hemoglobin (g/dL); HCT, hematocrit (%); MCV, mean corpuscular volume (fL); MCH, mean corpuscular hemoglobin (pg); MCHC, mean corpuscular hemoglobin concentration (g/dL)
b. Mann-Whitney’s U test, P< 0.001
c. Student’s T test, P<0.001
d. The murine strain shown in the front of each cross represents the high allele for phenotype
